# Diagnosis of a Rabbit Hemorrhagic Disease Virus 2 (RHDV2) and the Humoral Immune Protection Effect of VP60 Vaccine

**DOI:** 10.3390/cimb45080417

**Published:** 2023-08-08

**Authors:** Zhaoming Li, Kaimin Song, Yongzhen Du, Zhuanglong Zhang, Rupeng Fan, Pimiao Zheng, Jianzhu Liu

**Affiliations:** 1 College of Veterinary Medicine, Shandong Agricultural University, Tai’an 271018, China; 2 Research Center for Animal Disease Control Engineering, Shandong Agricultural University, Tai’an 271018, China

**Keywords:** RHDV2, RT-PCR, VP60, vaccine, immunity

## Abstract

Rabbit hemorrhagic disease (RHD) is known as rabbit plague and hemorrhagic pneumonia. It is an acute, septic, and highly fatal infectious disease caused by the Lagovirus rabbit hemorrhagic disease virus (RHDV) in the family *Caliciviridae* that infects wild and domestic rabbits and hares (lagomorphs). At present, RHDV2 has caused huge economic losses to the commercial rabbit trade and led to a decline in the number of wild lagomorphs worldwide. We performed a necropsy and pathological observations on five dead rabbits on a rabbit farm in Tai’an, China. The results were highly similar to the clinical and pathological changes of typical RHD. RHDV2 strain was isolated and identified by RT-PCR, and partial gene sequencing and genetic evolution analysis were carried out. There were significant differences in genetic characteristics and antigenicity between RHDV2 and classical RHDV strain, and the vaccine prepared with the RHDV strain cannot effectively prevent rabbit infection with RHDV2. Therefore, we evaluated the protective efficacy of a novel rabbit hemorrhagic virus baculovirus vector inactivated vaccine (VP60) in clinical application by animal regression experiment. The result showed that VP60 could effectively induce humoral immunity in rabbits. The vaccine itself had no significant effect on the health status of rabbits. This study suggested that the clinical application of VP60 may provide new ideas for preventing the spread of RHD2.

## 1. Introduction

Rabbit hemorrhagic disease is an acute infectious disease caused by RHDV. The disease could occur all year round. The incubation period is 48–72 h. The incidence and mortality of young and adult rabbits over 3 months are higher (up to 90%), which is transmitted through the respiratory tract, digestive tract, skin, and other ways. Systemic septicemia was even found in dead rabbits [1]. The virus first broke out in 1984 in Wuxi, Jiangsu Province, China, and other places [2]. And in a short period, about 140 million rabbits died of the disease, and the spread area was as high as 50,000 square kilometers, causing enormous damage to the country’s economy. Subsequently, the virus was also discovered in Italy in 1986 [3] and spread to other European countries, such as Spain and Portugal in the following year, causing serious damage to the local ecological environment [4,5]. In 1988, the first recorded outbreak in Mexico City, the Mexican government took strict clearance measures and became the only country to eradicate RHD. This measure also resulted in the death or elimination of approximately 110,000 rabbits [6]. The Australian government took the initiative to introduce RHDV to kill the hares they believed to be pests, resulting in the death of millions of hares [7,8]. In 1995, RHDV spread to other countries and territories. In 2000, the first report of rabbit plague appeared in the United States [9]. RHD has high infectivity, severe course, and a high mortality rate, with a high risk for the livestock industry [10,11]. Thus, RHD is included in the list of diseases of the World Organisation for Animal Health (WOAH), which was founded as the Office International des Epizooties [OIE]. The organization is subject to compulsory notification of every case, and an animal suspected or confirmed to have Lagovirus europaeus is immediately euthanized [12,13]. Nowadays, RHD spreads worldwide and causes enormous economic losses to the rabbit breeding industry [3].

The virus of RHD is endemic in most parts of the world, and genetic mutations in RHDV have also occurred. In 2010, a new strain of RHDV was first identified in France named RHDV2 [14]. In Portugal, Rouco et al. found that about one-third of the free-range European rabbit serotype has been converted from GI.1 to GI.2 [15]. In May 2015, RHDV2 (GI.2) was discovered in Australia; within 18 months, GI.2 spread to all states and territories of Australia and quickly became the primary circulating strain, replacing classical RHDV (GI.1) [16]. After, Taggart et al. analyzed more than 3900 rabbits in Australia within seven years and speculated that RHDV2 has three competitive advantages over RHDV: the ability to partially overcome immunity to other variants, the ability to infect young rabbits clinically, and a larger host range [17]. In 2020, China reported the first case of RHDV2 [18]. Currently, Singapore, Egypt, Ireland, America, Japan, and many other countries have recorded the emergence of RHDV2 [19,20,21,22,23]. RHDV is an icosahedral-non-enveloped virus. Both RHDV2 and RHDV are single-stranded positive-sense RNA viruses with similar genetic compositions. Their genomes are 7437 nucleotides in length and contain two ORF open reading frames. The longer ORF1 with 7034 nucleotides encodes multiple non-structural proteins p16, P23, P29, 2C-like protein, P15 (3C-like protein), VPg, virus protein-genome linked (VPg), P58 (RdRp) and a structural capsid protein viral protein (VP60), while the shorter ORF2 with 353 nucleotides encodes capsid protein VP10 whose function is not known [24,25]. The average nucleotide homology of RHDV2 and RHDV is 82.4%, and the homology between different strains of RHDV2 is about 99.3% [25]. RHDV2 is less virulent and less lethal than RHDV1, but with a longer course of the disease and increased virulence over time, with fatality rates varying between different strains of RHDV2 [26]. As RHDV2 has become prevalent worldwide, more and more strains have emerged that are as infectious and deadly as RHDV1 [17,26,27]. Therefore, it is essential to strengthen the effective prevention and control of RHDV2.

Currently, vaccination is the most effective way to prevent and control RHDV2. RHDV2 and the classical RHDV strain G1-G6 have similar typical clinical signs of death in rabbits, but there are significant differences in genetic characteristics and antigenicity [28,29]. According to reports, the vaccine prepared with the classical RHDV strain is ineffective against RHDV2 infection in rabbits [22,30]. RHDV2 vaccination could also lead to death from infection, with one report in the U.K. of pet rabbits dying within seven days of receiving the vaccine [30,31]. At present, there are few vaccines with good safety and high resistance to RHDV2, most of which rely on tissue-inactivated or attenuated vaccines for immunization [32]. This phenomenon brought a lot of trouble and economic losses to breeders and the rabbit industry system in China and even the world [33,34]. Therefore, finding an RHDV2 vaccine with solid safety and protection is necessary. 

The nucleotide diversity of the capsid protein (VP60) of RHDV2 differs from classical RHDV by more than 15%. As the main structural protein of RHDV, VP60 could self-assemble into virus-like particles (VLPs) in a baculovirus vector expression system. Therefore, the VP60 gene is an attractive choice for the detection of RHDV by Reverse-Transcription Polymerase Chain Reaction (RT-PCR) amplification and sequencing, as it is transcribed as sub-genomic RNA at high copy number during replication. It has the advantages of easy culture, expression, and post-translational protein modification [35]. According to the report, the vaccines made with VP60 and baculovirus vectors might have greater advantages over traditional attenuated or adjuvant inactivated vaccines in immunogenicity, vaccine production, and animal welfare [36,37]. However, the effectiveness of the specific VP60 vaccine is not clear.

In this study, we investigated a rabbit farm in Tai’an, Shandong Province. Approximately 1500 rabbits were present on the farm prior to the disease outbreak. Within ten days of the outbreak, 744 rabbits died, resulting in an average mortality rate of 49.6%. The rabbit farm promptly followed the regulations stipulated by the Animal Epidemic Prevention Law of China for the disposal of the affected rabbits during the outbreak. Then, we conducted clinical and pathological examinations, which showed classic clinical signs of rabbit hemorrhagic disease. The VP60 gene of the isolate was sequenced, and the results showed that the isolate was very similar to RHDV2. On this basis, we studied the effectiveness of a new commercialized rabbit plague baculovirus vector (VP60) vaccine against RHDV2. The challenge test and antibody detection were carried out, and the effect of the vaccine itself on rabbits was studied. The aim was to provide practical ideas for the clinical prevention of new rabbit hemorrhagic disease.

## 2. Materials and Methods

### 2.1. Materials

SteadyPure Virus DNA/RNA Extraction Kit, Evo M-MLV Plus 1st Strand cDNA Synthesis Kit, and ApexHF HS DNA Polymerase CL were purchased from Accurate Biotechnology (Hunan) Co., Ltd. (Changsha, China). IgG Ab and IgM Ab ELISA kits were from Shanghai Jianglai Biotechnology Co., Ltd. (Shanghai, China). Rabbit Viral Haemorrhagic Disease Baculovirus Vector Vaccine and Inactivated (Strain VP60) was obtained by Qilu Animal Health Products Co. Ltd. (Jinan, China). Gel Extraction Kit D2500 was purchased from Omega Bio-Tek (Norcross, GA, USA). The 35-day-old New Zealand white rabbits were provided by Qingdao Kangda Biotechnology Co., Ltd. (Qingdao, China).

### 2.2. Animals

New Zealand white rabbits, approximately 4 months of age, from an RHDV2-free commercial facility were assessed for general health. Rabbits were raised at 22 °C temperature and 50% humidity and fed with sterile food and water. The rabbits were randomly divided into a control group, classic vaccine group, new vaccine group, and classic + new vaccine group (30 rabbits in each group). The control group did not do any treatment. Each rabbit in the classic vaccine group and the new vaccine group was injected subcutaneously with 2 mL of the vaccine in the neck, and the classic + new vaccine group was injected subcutaneously with 1 mL of the two vaccines on both sides of the neck. Then, the temperature and weight of each group of rabbits were recorded every day within 7 days after the vaccine injection. At 7, 14, 21, 28, and 35 days after vaccination, blood samples were collected by cardiac puncture, and antibody detection was performed after the serum was obtained. 

### 2.3. Virus Used in Challenge

RHDV2 was discovered on a rabbit farm in Tai’an, China. The liver tissues (collected aseptically from freshly dead rabbits) preserved at −80 °C were repeatedly frozen, thawed, and ground three times, then pooled and homogenized in sterile phosphate-buffered saline (PBS) to obtain a 10% (*w*/*v*) suspension centrifuged at 4000 rpm/min for 10 min (low-temperature centrifuge, Hermle, Germany). The clarified supernatant was kept at −20 °C until use. Moreover, 2 mL of supernatant was subcutaneously injected into the neck of each rabbit, and deaths were recorded within 35 days after vaccine injection.

### 2.4. Histopathological Examination

The fresh tissues were soaked in 4% formalin fixative. Then, the tissues were soaked in gradient alcohol for dehydration and then embedded with paraffin. Moreover, 4 μm paraffin sections were stained with hematoxylin and eosin, and the stained sections were dehydrated with anhydrous ethanol, then washed with xylene and placed in a ventilation cabinet. Finally, the slices were covered with neutral resin and observed under an optical microscope.

### 2.5. Reverse Transcription-Polymerase Chain Reaction

The total RNA of the extracted samples was reversely transcribed into cDNA, and the cDNA was used as the template for PCR amplification of the RHDV2 VP60 gene. The reaction procedure was pre-denaturation at 94 °C for 1 min, denaturation at 98 °C for 10 s, annealing at 58 °C for 15 s, and extension at 68 °C for 100 s, with 35 cycles performed. At the end of the cycle, it was extended at 72 °C for 5 min and stored at −20 °C. Moreover, 10 μL PCR products were taken, and the target bands were observed by 1% agarose gel electrophoresis under the gel imaging system. DNA was recovered and purified by a gel recovery kit.

### 2.6. Phylogenetic Analysis

The phylogenetic analysis of VP60 gene sequences was performed using MEGA-X with the neighbor-joining approach on the Kimura two-parameter model. The reliability of the nodes was assessed with a bootstrap resampling procedure consisting of 1000 replicates.

### 2.7. Determination of Serum Antibody

Blood samples were collected by cardiac puncture at 7, 14, 21, 28, and 35 days after vaccination. After standing at room temperature for 1–2 h, centrifuge at 3000 rpm/min at 4 °C for five minutes to obtain serum. Then, follow the instructions of the ELISA kit (Shanghai Jianglai Biotechnology Co., Ltd.) to detect the serum antibody level. The absorbance was measured at 450 nm with a microplate reader. A standard curve was constructed based on the absorbance of the control sample to calculate the serum IgG and IgM content, respectively.

### 2.8. Temperature and Weight Measurements

During the whole measurement process, the room temperature was not changed more than 3 °C, and noise interference was avoided. The rabbits stopped feeding 2 h before the measurement and were placed in a suitable device until it was completed. The body temperature was measured with an anal thermometer with a precision of ±0.1 °C. The depth and time of inserting the anal thermometer into the anus were the same for each rabbit, the depth was about 1 cm, and the time was not less than one and a half minutes. The temperature was measured every 30 min, and the average of the two-body temperatures was recorded. The body weight was measured using an electronic scale, then recorded the data after the rabbit was quiet.

### 2.9. Statistical Analysis

All data were expressed as mean ± standard deviation (S.D.). GraphPad Prism 8.0 was used to draw the statistical chart. Statistical differences were evaluated by one-way analysis of variance (ANOVA) in SPSS Statistics 25, followed by Duncan’s multiple range test. Statistically significant differences were assumed at *p* < 0.05.

## 3. Results

### 3.1. Clinical Observation and Pathological Changes of Dead Rabbits

**Figure 1:**
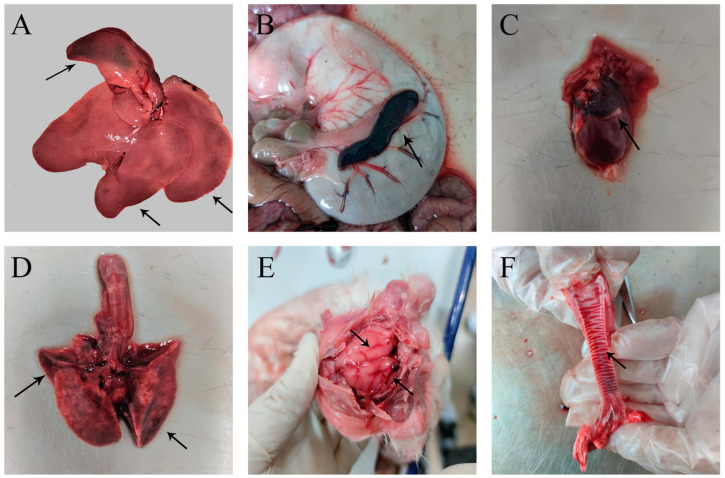
Clinical observation of RHDV2. (**A**) Liver enlargement and congestion, necrosis on edge, and bleeding spots. (**B**) The spleen is enlarged and dark purple. (**C**) Hemorrhage from the pericardium. (**D**) Lung congestion, with rice grain-sized bleeding spots. (**E**) Hemorrhage of the outer membrane of the brain. (**F**) Bleeding of the tracheal ring. The black arrows indicate the obvious lesions.

**Figure 2:**
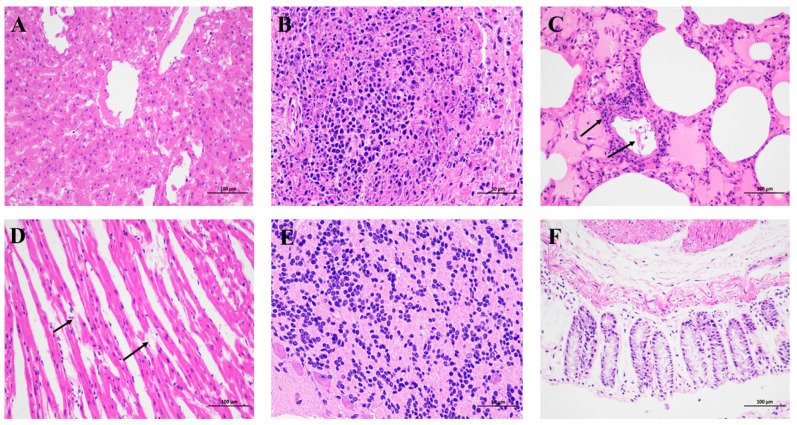
Histopathological changes of RHDV2. (**A**) Diffuse necrosis of liver cells (×200). (**B**) Lymphocyte necrosis of the spleen (×400). (**C**) Pulmonary interstitial edema, vascular endothelial cell shedding, peripheral lymphocyte infiltration (×200) (**D**) Heart interstitial edema and myocardial fiber breakage (×200). (**E**) Cerebral hemorrhage (×400). (**F**) Small intestine congestion (×200). The black arrows indicate the obvious lesions.

Before death, the rabbits showed struggling motion, prostration, dyspnoea, and occasionally bloody discharges from nostrils. In addition, we found that the clinical signs and histopathological changes of dead rabbits were very similar to rabbit hemorrhagic diseases through clinical necropsy and histopathological examination. From Figure 1A and Figure 2A, the liver showed swelling and hemorrhage, and the pathological results showed diffuse necrosis of liver cells. The spleen clinically showed dark purple and swelling (Figure 1B), and the pathological results showed lymphocyte necrosis (Figure 2B). We could see from Figure 1D that there were spotted hemorrhages in the lungs, and the pathological consequences of Figure 2C show pulmonary interstitial edema, vascular endothelial cells falling off, and lymphocyte infiltration around the blood vessels. Hemorrhage occurred in the pericardium (Figure 1C), and pathological sections showed interstitial edema and myocardial fiber breakage (Figure 2D). The hemorrhage was found in the meninges (Figure 1E and Figure 2E). Inflammatory cell infiltration was found in the epithelium of the small intestine (Figure 2F). Circular hemorrhage occurred in the trachea (Figure 1F). 

### 3.2. VP60 Genome Sequence and Phylogenetic Analysis of TA2020/0408 Isolated from RHDV2

**Figure 3:**
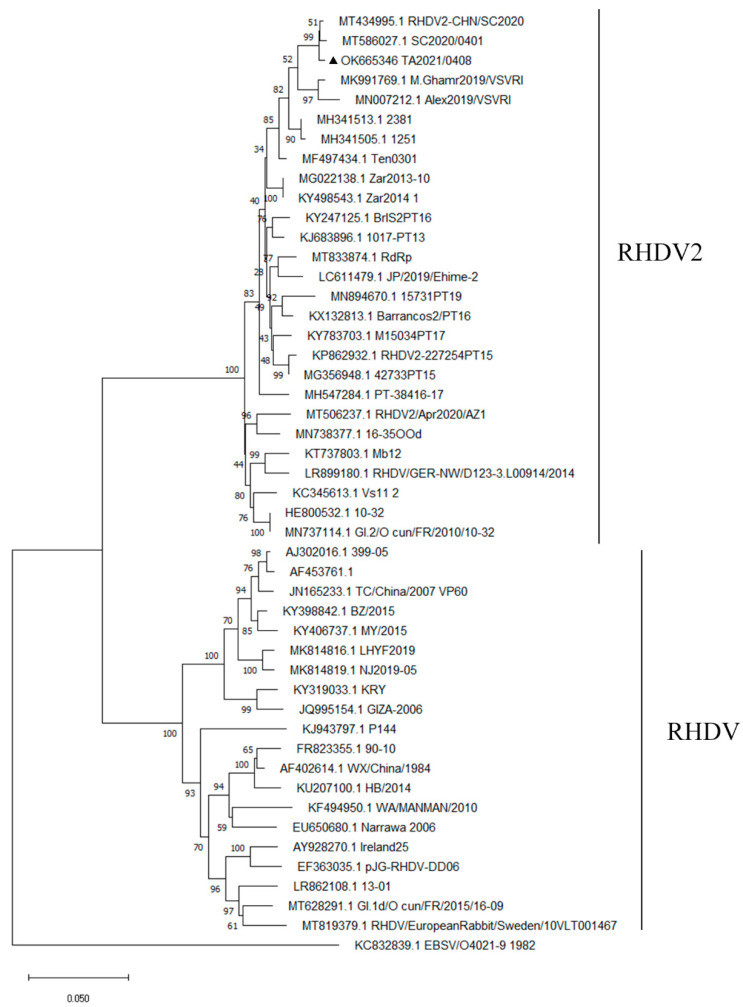
Phylogenetic neighbor-joining tree constructed based on the VP60 gene of RHDV2/TA/2020/0408 and multiple reference sequences. ▲ represents the isolated strain used in this study.

We designed primers based on the reported RHDV2 VP60 gene sequence (GenBank: HE819400.1) and used RT-PCR to detect RHDV2 VP60 RNA in liver tissue (Table 1). We cloned the VP60 gene amplified fragment into the PMD-18T vector for sequencing, obtained a 1636 nt genome sequence, submitted the genome annotation sequence to NCBI, and obtained the Genbank accession number OK665346. Then, we performed a phylogenetic analysis of the VP60 gene sequence obtained this time. Through investigation, we found that the isolate TA2020/0408 studied in this experiment and 26 RHDV2 strains were on the same cluster. The lowest nucleotide homology with the 2020 U.S. isolate RHDV2/Apr2020/AZ1 (GenBank: MT506237) is 94.7%, and the highest homology with the 2020 Chinese isolate SC2020/0401(GenBank: MT586027) is 99.3%. Compared with 20 classic RHDV strains, the homology is 81.2~82.5%. We have also compared a classic European brown rabbit syndrome virus, and the homology is 70%. It is worth noting that the isolate has 95.4% homology compared with the RHDV2 strain GI.2/O cun/F.R./2010/10-32 isolated in France in 2010 (GenBank: MN737114) (Figure 3). Compared with the partial nucleotide sequence of VP60 of the reference strain SC2020, the isolate obtained this time has three base changes, namely Met1829→Val1829, Thr2114→Ala2114, and Ser2153→Asn2153. We speculate that human activities may cause the long-distance transmission of RHDV2, and some genes may have been mutated due to changes in time and distance. The new isolate is in the same branch as the other RHDV2 strains. These results support the conclusion that the strain collected from Tai’an, China, in 2020 belongs to the RHDV2 (GI.2) gene group.

### 3.3. The Safety and Protection of the New Type of Rabbit Plague Virus Baculovector (VP60) Vaccine

**Figure 4:**
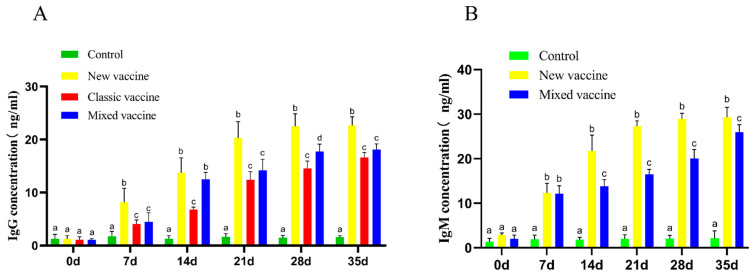
Antibody changes after vaccine stimulation. (**A**) The results of Ig G antibodies by vaccine stimulation. (**B**) The results of Ig M antibodies by vaccine stimulation. a, b, c, d were significantly different from each other, *p* < 0.05.

According to the antibody test results, the new rabbit plague vaccine has a good immunization effect, and the antibody concentration in the body continues to increase from day 0 to day 35. As shown in Figure 4A, the IgG antibodies of the rabbits in each group were all at a low level (below the negative cut-off point of ELISAs) before vaccination (day 0). The IgG concentration of the new vaccine group was significantly higher than that of the control group and the classical vaccine group at 7, 14, 21, 28, and 35 days after vaccination and considerably higher than that of the mixed vaccine group at 7, 21, 28, and 35 days after vaccination. Figure 4B showed that IgM concentration in all groups was low before vaccination without any difference. IgM concentrations in the new vaccine group were significantly higher than those in the control group at 7, 14, 21, 28, and 35 days after vaccination and substantially higher than those in the mixed group at 14, 21, 28, and 35 days after vaccination (*p* < 0.05). It could be seen that the new rabbit plague vaccine can effectively stimulate the body to produce antibodies and have a protective effect on it.

To verify the reliability of the new RHDV2 vaccine, we conducted the RHDV2 challenge experiment (Table 2). The results showed that the survival rate of rabbits immunized with the VP60 vaccine was 93.33% (14/15) after 48 h of challenge, while the survival rate of rabbits unimmunized was 0 (0/15). Compared with normal rabbits, vaccinated rabbits did not show changes in pathology and histopathology after the challenge, and RT-PCR did not detect the spread of RHDV2 in blood and tissue organs. We concluded that the VP60 vaccine had high protection against RHDV2 after 35 days of inoculation.

**Figure 5:**
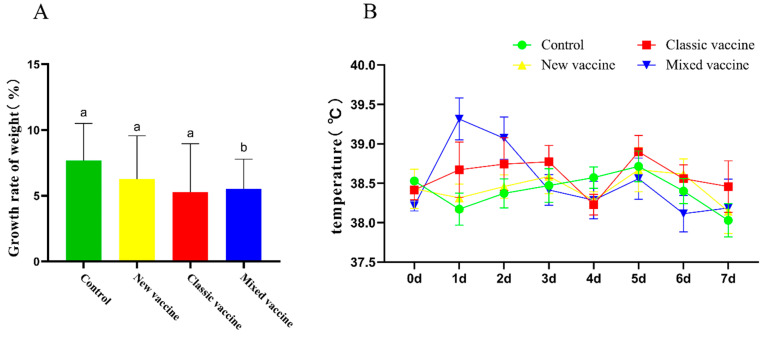
The results of the body temperature and weight of rabbits in this experiment. (**A**), The growth rate of body weight within seven days after vaccination. (**B**), Changes in body temperature 7 days after vaccination. a and b were significantly different from each other, *p* < 0.05.

By measuring the body weight, we found that 7 days after immunization, the weight gain of rabbits in the new vaccine group and the classic vaccine group was insignificant compared with that of the control group. Still, the weight gain of the mixed vaccinated rabbits was smaller than that of the control group (Figure 5A). We speculated that the individual vaccine immunization has little effect on the eating of rabbits, and mixed vaccination may cause the body weight to increase slowly in a short period. Within 7 days after immunization, the rabbits in the mixed vaccination group showed a significant increase in body temperature within 1–2 days after vaccination. In the other two vaccine immunization groups, the body temperature of individual rabbits exceeded 39 °C (Figure 5B). However, we believe that this situation is normal after the vaccination. 

## 4. Discussion

Since the 1890s, rabbit hemorrhagic disease has spread all over the world. As a highly infectious and fatal disease, it has caused great damage to the rabbit breeding industry, the ecological environment and the national economy [3]. In 2010, France first reported a new genotype called RHDV2, which has a wider host range than classical RHDV, which means that RHDV2 is more destructive [38]. At present, RHDV2 is gradually replacing RHDV1, and the prevention and control of the epidemic have become a global problem [39]. 

In the case of RHDV2 discovered by Toh et al. in Singapore, the liver had a severe acute liver injury, diffuse necrosis and degeneration of liver cells, and many inflammatory cell infiltrations [19]. Hänske et al. found that pet rabbits infected with RHDV2 had congestion of the trachea, interstitial pneumonia, and chronic enteritis [32]. Miao et al. found in the Netherlands that infected European rabbits had acute liver necrosis, splenic necrosis, and acute pneumonia [40]. These are very similar to our clinical findings. There are also many reports of clinical signs such as anorexia, lethargy, reluctance to move, pyrexia, tachypnoea, and jaundice in cases of natural infection [1,21]. Since our RHDV2 challenge is carried out by subcutaneous injection, the course of the disease progresses rapidly. We observed the fact that the rabbits showed struggling motion, prostration, dyspnoea, and occasionally bloody discharges from nostrils. Our necropsy and histopathological examination confirmed that the rabbit killed by RHDV2 infection had typical RHD characteristics.

In this study, the RHDV2 VP60 gene was obtained from the liver of infected rabbits; then, we performed a phylogenetic analysis of the VP60 gene sequence. Through investigation, we found that the new isolate is in the same branch as the other RHDV2 strains. The nucleotide homology analysis revealed that RHDV2 isolates in China and Europe were closely related, which may indicate rapid growth transcontinental transmission of the virus and support the conclusion that the strain collected from Tai’an, China, in 2020 belongs to the RHDV2 (GI.2) gene group. In addition, viruses often recombination [41,42,43]. In the first case of RHDV2 reported in Singapore, the researchers found that the whole genome sequence of the obtained isolate (Genbank: MW194928) has 90.05% and 87.7% homology with the rabbit calicivirus sequence (Genbank: X96868.1) and the Algarve1 RHDV2 sequence (GenBank: KF442961.2), respectively. They suggest that this result may be caused by recombination between RCV and RHDV2 [19]. At the same time, some researchers speculated that RHDV1 and RHDV2 have co-circulation and are prone to a mutation in the circulation process, which also brings many difficulties to the prevention and control of RHDV2 [18].

There was a strong demand for effective vaccines to protect rabbits against this disease since, in 1984, RHDV1 killed millions of rabbits in commercial husbandries in China [2,44]. However, because of the marked antigenic difference between the classical RHDV and RHDV2, rabbits vaccinated against the classical RHDV strains were not completely protected against RHDV2 strains [45]. In this work, we show a new type of rabbit plague baculovirus vector (VP60) vaccine that could protect against RHD caused by RHDV2. Reemers et al. used a novel trivalent myxomatosis virus vector RHDV vaccine. They demonstrated simultaneous immunity against myxomatosis virus, RHDV1, and RHDV2 viruses, noting that the vaccine had no adverse effects in pregnant rabbits and showed a good immune response [46]. Wang et al. [47] used recombinant Lactobacillus casei expressing vp60 of RHDV to produce a vaccine that can immunize orally and was safe and effective in inducing mucosal immunity. Omar Farnós et al. [48] used the high expression capacity of Pichia yeast to recombinantly express RHDV vp60 to protect mice from RHDV. Qi et al. [36] used Baculovirus expression vectors to express vp60 of RHDV and RHDV2 and produced a bivalent vaccine that protects rabbits against RHDV. Müller et al. developed a recombinant RHDV2-VP1 vaccine using a recombinant baculovector and proved that the vaccine could protect rabbits, significantly reduce viral protein replication level and limit viral shed, ensuring the safety and effectiveness of the vaccine [45], which was very similar to part of our experiments. It has also been reported that very low levels of viral RNA were detected in inoculated rabbit livers, but no viral replication or virus particles were found [49]. Baculovirus expression vectors are easy to culture, express proteins, and mature in mass production, so they are widely used in laboratories or factories to produce VLP [50]. Our study showed that rabbits inoculated with the novel rabbit plague virus baculovector (VP60) vaccine could effectively resist RHDV2 challenge after 35 days. It is important to note that RHDV cannot be cultured in vitro, and most RHD vaccines were made from the liver of infected or diseased rabbits, which is not consistent with animal welfare [45]. The vaccine we used in this study is a new commercial RHDV2 vaccine developed using RHDV2 VP60 capsid protein expressed in the baculovector system, which is essential for animal welfare. In order to conduct a more comprehensive study on the performance of the new vaccine, in future studies, we will incorporate relevant experiments to compare the immune response duration of the two vaccines and transmission electron microscopy experiments on the protein related to the new vaccine.

## 5. Conclusions

In this study, we conducted clinical and pathological examinations on a rabbit farm in Tai’an and found the typical clinical signs of rabbit viral hemorrhagic disease. We sequenced the VP60 gene of the isolate and found that the isolate was very similar to the SC2020 gene sequence through the phylogenetic tree, indicating that the cause of this disease was caused by RHDV2, which may have spread over long distances in China. On this basis, we explored the effectiveness of the new rabbit plague baculovirus vector (VP60) vaccine against RHDV2 through challenge experiments and antibody detection and studied the effect of the vaccine itself on rabbits. It was proved that the VP60 vaccine showed a strong protective force against RHDV2 attack 5 weeks after vaccination and could effectively induce humoral immunity in rabbits. When used alone, it is safe and effective for rabbits. Our purpose is to provide practical ideas for the clinical prevention of new rabbit hemorrhagic diseases.

## Figures and Tables

**Table 1:** Primers used for quantitative real-time PCR.

Primers Name	Gene Segment	Primers Sequence (5′→3′)	Product Size (nt)
VP60 F/R	VP60	F: atggagggcaaagcccg	1636
		R: tcagacataagaaaagccattagttgtgcc	

**Table 2:** The results of survival rate data of novel rabbit plague baculovector vaccine against RHDV2.

Group	Quantity	Challenge after 2 Days	
		Survived	Died
Vaccinated	15	14	1
Unvaccinated	15	0	15

## Data Availability

The data shown in this study are contained within the article.
